# Shifting parental roles, caregiving practices and the face of child development in low resource informal settlements of Nairobi: experiences of community health workers and school teachers

**DOI:** 10.1186/s12991-018-0219-x

**Published:** 2018-11-26

**Authors:** Manasi Kumar, Beatrice Madeghe, Judith Osok-Waudo, Grace Nduku Wambua, Beatrice Kagai Amugune

**Affiliations:** 1 Department of Psychiatry, University of Nairobi, Nairobi, 00100 Kenya; 2 Department of Psychology, University College London, London, WC1E 6BT UK; 3 Department of Food Science Nutrition and Technology, University of Nairobi, Nairobi, 00200 Kenya; 4 Department of Clinical Neuro- & Developmental Psychology, Vrije University of Amsterdam, Amsterdam, Netherlands; 5 School of Pharmacy, University of Nairobi, Nairobi, 00200 Kenya

**Keywords:** Parenting practices, Child development, Teachers and community health workers perspective

## Abstract

Approximately, 42% of the Kenyan population live below the poverty line. Rapid growth and urbanization of Kenya’s population have resulted in a changing poverty and food security environment in high-density urban areas. Lack of basic food needs in Kenya affects approximately 34.8% rural population and 7.6% of its urban population. Using multi-community stakeholders such as teachers and community health workers (CHWs), this paper examined food insecurity and its consequences on caregiving practices and child development. A qualitative study design was utilized. Key informant interviews and focused-group discussions with four primary school teachers and three CHWs and a nurse in-charge working within Kariobangi and Kangemi were applied to elicit various perspectives from family-, school- and community-level challenges that influence caregiving practices and child development. Grounded theory method was applied for qualitative data sifting and thematic analysis. Our findings exposed various challenges at the school, family and the community levels that affect caregiving practices and consequent child development. School-level challenges included lack of adequate amenities for effective learning, food insecurity, absenteeism and mental health challenges. Family-level barriers included lack of parenting skills, financial constraints, domestic violence and lack of social support, while community challenges such as unemployment, poor living conditions, cultural practices, lack of social support and poor community follow-up mechanisms contributed to poor parenting practices and child development. Parenting practices and holistic child development strategies in resource poor settings should focus on parenting skills, food security, quality education and addressing parents and children’s mental health challenges.

## Introduction

In Kenya, 13.4 million Kenyans live below the poverty line, comprising 52.3% of the rural population and 34.8% of urban dwellers living in poverty [1]. Poverty index stands at 36.1% based on the Kenya integrated Household Budget Survey 2015/16 [2]. Extreme poverty defines that basic food needs are not being met even when all resources are devoted to food and this affects nearly 34.8% of the rural population in Kenya and 7.6% of its urban population [3]. Research evidence indicates that the concentration of poverty and malnutrition is now shifting from rural areas to urban areas.

Income-related food insecurity has been linked to a multitude of negative physical and mental health conditions; they suffer from higher rates of disease and overall poorer health [4]. It has been found to lead to parental depression which has a detrimental impact on parenting [5, 6]. Parental depression negatively affects fathers’ and mothers’ caregiving, material support, and nurturance, and is associated with poor health and developmental outcomes for children of all ages [6]. Food insecurity influences health and development through its effects on nutrition and as a component of overall family stress [5]. Research has found a link between child malnutrition and depression that mothers with depressive symptoms were 40% more likely to have underweight or height-stunted children than mothers who were not depressed [7]. These children are also at increased risk for a range of poor social, emotional, neurocognitive and behavioral outcomes [8].

Apart from poor parenting skills demonstrated by food insecure-depressed parents, research has shown that a parents’ own childhood experiences of maltreatment are responsible for up to one-third of the variance predicting child maltreatment [9, 10]. Child abuse and neglect in Kenya is a serious public health problem, with findings indicating that during childhood, 32% of females and 18% of males experience sexual violence, 66% of females and 73% of males’ experienced physical violence, 26% of females and 32% of males experience any violence as a child and 13% of females and 9% of males experienced all three types of violence during childhood [11]. According to Mbagaya et al. [12], the prevalence of child physical abuse without differentiation of its severity in Kenya is 59%. The KVACS [11] report shows that violence against children was mostly perpetuated by parents or close relatives. Economic hardship has been linked to harsher and less responsive parent–child interactions, with overall adverse outcomes for children [13]. Studies on the relationships between parenting, household poverty, and child neglect found that parental perceptions of material hardship and infrequent employment predict child physical neglect, as also do a lack of fun activities and praise for the child, spanking, and frequent television viewing [1]. When adults are hungry, they display worse parenting, and that when adults have current parenting difficulties their children are more likely to be experiencing hunger [14].

Using surveys conducted in 2003, 2008–2009 and 2014 by Matanda et al. [15] found that optimal childcare practices in Kenya were significantly correlated with area of residence (region), household wealth, maternal education, parity, mother’s age, child’s age and pregnancy history. To understand family-level and parenting challenges that influence child development, one has to look at them from multiple angles. Golden et al. [16] recognised the development of individuals within nested environmental subsystems/contexts addressing the complexities and interdependences between socioeconomic, cultural, political, environmental, organizational, psychological, and biological determinants of behaviour. A gap exists in the between the ideal and reality of basic childcare in the home. Kenya has several notable programs aimed at improving the health and well-being of children [15]. These programs highlight the importance of and inclusion of multiple stakeholders to ensure the well-being of the child. It is with this in mind that this study aimed to gain a better understanding of the role of shifting parental roles and caregiving practices in the development of children from the view of the teachers and health workers in two low-income residential estates in Nairobi, Kenya. Their views can inform what parents are going through and what knowledge, service and support barriers exist.

## Methods

We received ethical clearance from the Kenyatta National Hospital and University of Nairobi Institutional Review Board no. KNH/UoN-ERC Ref. P520/08/2015. Using a qualitative approach, we carried out two focus group discussions with teachers from three schools located in Kangemi informal settlements and community health workers. We carried out one face-to-face facility with the in-charge from Kariobangi Health Centre between July and August 2016. The teachers were four females who had been in the profession for over 15 years and were involved with lower and upper primary education within Kariobangi area. The Community health workers (CHWs) included one male and two females, and the sister in-charge at the Kangemi health centre.

The key informant interviews (KIIs) were 35–45 min long and the focus group discussions (FGDs) were 1–1.5 h each carried out at the respective health facilities (Table 1).

**Table 1:** Interview domains made to the teachers and community health workers

Teachers *n* = 4	Community health workers and facility in-charge *n* = 4
What are your observations about child behavioral problems in schools? How do you think the children cope with problems? What is the source of these problems? What do you think are the challenges at home? How are parents coping with the child behavioral problems? What is parents’ awareness and knowledge about parenting?	
What do parents need?	What are the needs of teachers and community in addressing parenting challenges?
How can community health workers and other health workers help families and be supported in their work?	How can the teachers be better supported?

## Data collection

In total, one key informant interview and two focused group discussions with four primary school teachers and three community health workers we carried out. The teachers and health workers were invited to participate in the study based on their interest in maternal and child mental health and parenting practices. They had not received any training in mental health. The interviews were audio-recorded with consent from the participants. The interviews were conducted in Kiswahili and English.

## Data management and analytical approach

All FGDs and KIIs were transcribed in English and deidentified. The research team (BM, JO, MK and GNW) independently, and using both deductive and inductive analysis approaches, first coded the data based on our assumptions/research questions. Thereafter, we used inductive approach where we reviewed the data and begun to take note of emerging themes and patterns based on other contextual insight and we began to interpret and draw conclusions. The codes were discussed and categorized to further establish the inter-coder agreement. The most relevant themes were discussed and those that were reoccurring were collated together.

## Results

The following analysis organizes the perspectives of teachers and health workers on the number of issues that families and community face in relation to parenting and child development issues. For organizational purposes, we presented findings in two sections. The first section represents findings from teachers; the second section represents findings from community health workers and facility in-charge.

### ‘We have to mould the children’: perspective of the teachers

All four teachers unilaterally talked about how despite overcrowding, limited infrastructure, support and training; teachers have had to take up the role of ‘molding the children’ as parents were drunkards, while others struggling for livelihood and were, therefore, unable to take parental and caregiving responsibilities seriously. There was also child abuse and incest in families and in the communities that the teachers talked about and how they had to direct efforts towards making their pupils aware of these and at times they receive support to hold talks and demonstration session from community health nurses.

*‘Like the current parent is lacking the molding skills…..so you find that the children have not been molded from the word go …..so it’s like this being a school ……we are also trying as much as we can …..to mold the children ….. because most of the parents…most of them being drunkards …… neglecting their children ……so you see now the burden has been placed upon the teacher …. It’s the teacher teaching, the teacher is doing parenting and molding.’*

The teachers talked about the impact of early pregnancy in their communities where children were having children—and, therefore, the very young mothers and parents often do not know enough about caregiving. And the caregiving task became their responsibility in some ways.

*‘You find children…children give birth to children …..so they lack the knowledge and skills….. most of them they don’t know how to handle their children ……so it means this burden has been thrown back to the teacher.’*

Another theme that the teachers brought out was the impact of cultural practices on the child’s development and a teachers’ sense of satisfaction. One of the issues they all highlighted was that due to high food insecurity in the community, the families struggled to make ends meet. In this struggle, the caregivers often were unable to address the child’s health issues in time until things worsened. The child’s absence from school was a persistent issue and impacted on the teachers work with the child. If this was a specifically weak pupil or unusually bright one, their prolonged school absence impacted not only their performance but also the teachers sense of satisfaction in carrying out their work effectively.

“*There are cultural issues …..one of our problem is …because you find that there are some communities that if a member of family dies…. they’ll have to…they have to stay home for some …some months….and this child is expected to be in school ….so you find that as you move on with the syllabus …..there is this kid who is lagging behind”.*

Another important issue they brought out was that food insecurity was a challenge and that the parents were struggling to make ends meet. The teachers’ offered food to top-up whatever the child may have had (many times it is one meal per day) as part of the school feeding program. They were aware that the child may be active and receptive for learning for limited time if the hunger persists or if the child is unwell and this is a constraint they have learnt to appreciate and work with. The school asks for an annual fee from parents to support the feeding program but international NGOs supports this initiative on and off. There was an added difficulty earlier when the NGOs would buy food items but currently they would remit payment to the school to procure all necessary materials and this process suffers from delays.

*“….. Another problem is about what…health diet you find these children now like now we give them food here in school githeri they take githeri …some of them can come …that one home they eat and then again tomorrow ….so they come very hungry in school …they don’t have anything; and you know that food is now cold … when they eat it can affect their what…their health … so most of them are really sick; they are weak; you know they are young …. they are young …. they eat without washing their hands”.*

“*Children in the locality around there’s several challenges I may say because the…the…the community around is poverty stricken as you know that ….and because the social class is very low …..parents tend to suffer so much …. because they look for money here and there and wherever ….. most of them are not employed …..so children who come from these families really face these challenges; whatever happens with the parents …..falls back to the children ….and these are…you find that at times a child comes to school …..without taking breakfast …..a child can go without supper …… most of them even depend on the meals we give here in school*”.

Two teachers alluded to the extra sensitivity with which they handled a child living with HIV. They said that part of their training prepares them with basic guidance and counseling which has made them aware of the need for extra care and confidentiality of children with illnesses or problems such that social stigma could be minimized. The teachers shared that in each of their class, on average, 2–3 children were living with HIV.

“*Parenting issues ….it is really…it is really challenging with the community around ….. because most parents here are single parents ….. some of them are HIV affected …. as in the other partner maybe was infected and passed on …. or still infected and the other one is nursing … and we even have children who are infected with HIV and AIDS.. and we manage them because when a parent comes he/she explains he/she opens up and tells you this is the condition ….. and then from there we pick up. There are those who come who are single*-*parented we have to know…There are those who are abused …. yes; psychologically, sexually, physically and we experience all these. Personally I have; because apart from being a teacher I am interested in counseling ….and I face them. And when they come we handle them the way they come.”*

Part of their intervention with the child who is experiencing distress also extends to his/her parents to provide skills and support. If there are instances of parental discord or violence at home which they get to know and feel impacts the learning of the child, they then invite the parents to come in and discuss the issues with them. The teachers categorically stated that their concern was to address the child’s learning. If there were sexual abuse and severe maltreatment issues, they may hand over the cases to the children’s rights officer for further review and parental surveillance. There were cases where as teachers they do not feel secure enough to visit and carry out home visits and in such cases, the child protection officer is, in those instances, the more appropriate person to intervene.

*‘You see some matters we…we go to an extent of involving the parents ….. because whereby you’ll find a child who is absent ….. for 3…4* *weeks ….. then you have to dig into the child to know what is really happening. So the child will just expose that “you see my father and my mother are doing A, B, C, D that is affecting my… learning” ….so you have to come in now as a teacher but now you sit in a position of…of a counselor and try to bring the two together …..or if there is violence in the home …… you can also talk to them ….for the sake of this child.’*

*‘The family lacks space and awareness around protecting the children from undesirable exposure. One child told me ….my mom and dad were doing raping……I was shocked at the repeated mention of the word raping ….until I called the parent; the mother to ask her…. what did you do? As the child has told me that you were doing raping and she explained that they were intimate and the house is very small and as they are doing their own things and the child…you know, sees this and is petrified by it.’*

*‘Now domestic violence even affects the children when father decides to fight the mother and children ….send them away ….those ones happen. There are those where a father feels he married a mother with a big child …..and the child is not his … so he ends up abusing the child sexually or even physically. Like last year we had such a case; a candidate was beaten seriously with a step father ….. because the mother was just married to him just a year before ….. so the girl was just a big girl; and the girl knew everything ….. but because the father did not give consent ….. that the girl is his …. he was using the girl as a house help …. so when the girl wanted maybe to explain her rights ….. he decided to beat her and send away.’*

Children with mental illness and developmental disorders attend their classes without any resource or support to the child or the teachers. Our participants mentioned that in overcrowded classes, it becomes challenging to deal with children in need of special attention. The teachers mentioned that the parents do not understand or accept that their child may be autistic or have intellectual impairment. The teachers consequently had to maintain them in regular classes where they were not able to support the children.

*We have several children who have learning disabilities or are mentally challenged….we also have those with autism…you know when you tell a parent…. the child is like that … they don’t accept so we keep on having them in class. The overall environment is affected and of course this child learns very little.”*

### ‘No body prepares them for what a family needs to do for each other’: perspective of the community health workers and the health facility in-charge

The nurse in-charge and community health workers allude to the need for support groups to address maternal mental health in the community. She said that those groups also applied to parents in general to normalize their experiences, and for parents and caregivers to address the problems jointly. The health facility as per the nurse in-charge was not able to support participation of men and women (partners or families together) which presented a great barrier to care. She thought it was critical to engage with men as fathers and husbands to address violence, their own stress and care.

*“….I usually encourage them, even when they come with their.. as couples we give the first priority …. and then we tell the other mothers, ‘next time come with your partner it will be better when we are talking with the partner…the partner instead of one on one’ (‘that go and tell your husband to buy you this, to do this,’ I don’t know whether the husband has money….so when they are there the husband will say ‘I am supposed….I’m able to do this and this and if I get finances I will be able to do what you are……what I am supposed to do. So it’s better when they are in that couple” (facility in*-*charge).*

It was also emphasized that once there are needy children or parents especially those living with HIV or malnourished or adolescent parents, once they come to the health facility through support from the community health workers, they try their best to keep a track of them and follow them up regularly. However, there are challenges when parents face greater problems including if the mother is alcohol dependent, unwell or in abject poverty and unable to reach out to the health facility. These cases present the most challenge.

The community health workers articulate that they are a buffer between parents and families in the community and the health facility. They know that there are some issues that the parents need encouragement and support to bring before the nurses and doctors. One male CHW mentioned that parents are daunted by the financial implication associated with visiting the health facility in terms of transport, treatment costs and there is the cost associated with time taken in queuing up for hours losing one’s livelihood. And if this is a single mother, then it becomes more important to fend for the family than to get herself or the child seen first. This male CHW also articulated the missing father in the parenting context, indicating that building greater support and inclusive approach for engaging with fathers is critical.

“….*men…men are very much left out in…in (in terms of programs… … in terms of engagement….. it’s men…men are left out … they don’t know much about child upbringing into aahh…. Whatever the child … how many immunizations they should have ….in what time they should come to the…ee…ee to the clin….to follow up for the clinics you see (they don’t know a lot …..….; they need to know …… to be educated…..they also have their own issues that need to be looked at”.*

One older female CHW talks about the kind of support she offers parents, especially mothers, to help them find livelihood to take care of the family needs. In her own words

“*it so happens because sometimes you find it’s a mother who is alone; has school fees burden so we sit down with her and talk to her; we tell her if there is something she can start doing so as to get even a shilling; we advise her if she doesn’t have capital we tell her to start looking for it; we even tell her there are women who wash people’s clothes for cash; so she goes to wash for people gets sh. 50, keeps 10 and uses 20 in a month’s time she has capital; she can start selling tomatoes to help herself. Or if we know of somewhere to find help yet she doesn’t; we refer her and goes to get help.. ehh”.*

This kind of encouragement and direction helps a family feel like they are together instead of leaving them starving and desperate. The first parenting intervention is to provide parents resources to feed and look after their children.

Another help they provide to the community is to connect parents with problem behaviors or adolescents with substance abuse or irresponsible behaviors to the chief or elders in the community. Finding ways to bring a problem behavior to the attention of community authorities including district courts in case of abuse or disruptive behaviors, child protection officers when there is sexual abuse or negligence or health problems to health facility are pathways the CHWs talked about which they follow in their routine practice.

## Discussion

Our study tried to tap into the everyday practice and experience of teachers and health workers in the community to understand challenges around parenting and caregiving and the broad implications for child development.

Our study participants pointed to a number of stressors and adversity experienced by families living in informal settlements. The families lived in stress that emanated from poverty, unemployment or limited income and a social environment that had plenty of negative influences like substance abuse, alcohol, violence and poor social support networks for families. Parental investment on children was hindered by poverty. Urbanization has brought other changes that some pupils stayed with their grandparents and the parents may have migrated to other cities for better economic prospects and provide for the family from there. In such scenarios, they act as parents to make the everyday learning and nurturing for these children. Research has found the role of grandparents in the raising of their grandchildren as a positive resource; where they contribute to the grandchildren’s schooling and play a strong role on their upkeep [17].

Parenting and family environment are supposed to buffer the impact of instability on the child, but our findings have shown that this is not the case, hereby showing us that instability weakens the quality of parenting and the home environment, thus negatively influencing the development of the child [18]. Powers [1] and Sandstorm and Huerta [18] state that instability in the child’s environment as evidenced by employment instability of the parent impacts the economic stability of the family, which in turn influences parenting and the family environment. The teachers provided us with insights around parental handling and engagement with their children. Both shared scenarios where parents were negligent or maltreated the children, leaving the teaching with the role of providing appropriate parenting when the caregivers were unable to.

Interpersonal violence was a significant theme not only between caregivers in the informal settlements where life was very haphazard but also there were cultural practices of FGM in some communities, maltreatment from step-parents to single mothers who were unable to meet needs of multiple children. The gender norms in our findings suggested that the fathers were more disengaged and the pressure of caregiving was left on the mothers. There were many single mothers who juggled between fending for the family and attending to educational and health needs of the family. Research has highlighted the importance of fathers to the well-being of the child, stating their involvement fosters healthy child development [19]. One health worker pointed to the need to engage with fathers and men in the community. She felt that men engage in interpersonal violence with their partners and remain disengaged with parenting as they have not been given skills and that space in the family to discuss emotions and family well-being. This health worker also reiterated that by engaging with men we can also convey the message that parenting is not a maternal task alone—it is something that both parents need to share. By sensitizing men to their role as fathers, families could be better supported.

The interviews left us with the feeling that these families needed psychosocial support in forms of parenting support groups, support groups for couples and general hands-on skills and awareness in the community around supporting families who live on very little. Programs aimed at improving parenting skills which can contribute to better well-being of the child, recommendations on parenting programs that include and engage fathers from a respectful and cultural stand point would be welcome.

It appeared that the teachers and health workers felt very overwhelmed in their additional family support roles. They were ill-equipped resource-and-skills-wise to support children and parents who had challenges. Awareness programs for parents were recommended by both set of our participants who also mentioned that school could be a point of entry to engage with parents. The formation of support groups to be offered at either health facility or in schools needed a cadre of teachers who were well-trained in psychological first aid, parenting or simple group interventions. Both health workers and teachers alluded to mental health care for young mothers and parents as well as intervention targeting fathers. The dual engagement of health workers and teachers in the service of attending to parenting needs and empowerment of urban poor parents struggling with livelihood is critical.

## Conclusion

Understanding parenting and child development through the eyes of teachers and health workers provided unique insights. We identified several family-, community-, school- and health facility-level gaps and challenges that are need redressal. In many resource constraint settings, parenting is thought to be an automatic skill that comes by itself rather than a capability to be harnessed in caregivers, families and the community. The teachers and community health workers shared their understanding of parenting challenges which need to be addressed in a holistic community response to mental health.

Study findings also highlight the essential benefits of sustainable school food supplementary programs in resource poor settlements and sensitization of parents/caregivers on the effects of parental practices on child development and wellbeing.

## Abbreviations

AIDS: acquired immuno-deficiency syndrome
CHW: community health workers
FAO: Food and Agriculture Organization
FGDs: focus group discussions
FGM: female genital mutilation
HIV: human immunodeficiency virus
KII: key informant interviews
KIHBS: Kenya Integrated Household Budget Survey
KNBS: Kenya National Bureau of Statistics
KVACS: Kenya Violence against Children Survey
NGO: Non-Governmental Organization (s)
